# Acute Stroke With Large Vessel Occlusion and Minor Clinical Deficits: Prognostic Factors and Therapeutic Implications

**DOI:** 10.3389/fneur.2021.736795

**Published:** 2021-10-22

**Authors:** Bastian Volbers, Rebecca Gröger, Tobias Engelhorn, Armin Marsch, Kosmas Macha, Stefan Schwab, Arnd Dörfler, Stefan Lang, Bernd Kallmünzer

**Affiliations:** ^1^Department of Neurology, University of Erlangen-Nuremberg, Erlangen, Germany; ^2^Department of Neuroradiology, University of Erlangen-Nuremberg, Erlangen, Germany

**Keywords:** mechanical thrombectomy, minor stroke, large vessel occlusion (LVO), acute management of stroke, outcome

## Abstract

**Background and Purpose:** The optimal acute management of patients with large vessel occlusion (LVO) and minor clinical deficits on admission [National Institutes of Health Stroke Scale (NIHSS) ≤ 4] remains to be elucidated. The aim of the present study was to investigate the prognostic factors and therapeutic management of those patients.

**Methods:** In this retrospective cohort study, we investigated (1) all patients with acute ischemic stroke due to an LVO who underwent mechanical thrombectomy (MT) and (2) all patients with minor clinical deficits (NIHSS ≤ 4) on admission due to an LVO between January 2013 and December 2016 at the University Medical Center Erlangen. We dichotomized management of patients with minor deficits treated with MT for analysis according to immediate mechanical thrombectomy (IT) and initial medical management with rescue intervention (MM) in case of secondary deterioration. Primary endpoints were secondary deterioration, in-hospital mortality, and functional outcome on day 90 (dichotomized modified Rankin Scale 0–2: favorable, 3–6: poor).

**Results:** Two hundred twenty-three patients (83% with anterior circulation stroke, 13 (6%) with minor deficits) treated with MT and 88 patients with minor deficits due to LVO [13 (15%) treated with MT] were included. Secondary deterioration (*n* = 19) was independently associated with poor outcome in patients with minor deficits and LVO [odds ratio (OR), 0.060; 95% confidence interval (CI), 0.013–0.280], which in turn was associated with the occlusion site [especially M1 occlusion: 11 (58%) vs. 3 (4%) in patients without secondary deterioration, *p* < 0.0001]. IT (*n* = 8) was associated with a lower intrahospital mortality compared to MM (*n* = 5; 13 vs. 80%; OR, 0.036; 95% CI, 0.002–0.741). Seven of eight patients with IT survived until discharge, with 29% showing a favorable functional outcome on day 90.

**Conclusions:** Secondary deterioration is associated with poor outcome in patients with LVO and minor deficits, which in turn was associated with occlusion site. Future randomized controlled trials should assess whether selected patients, depending on occlusion site and associated characteristics, may benefit from MT.

## Introduction

Recently, a series of randomized controlled trials (RCTs) could demonstrate that endovascular mechanical thrombectomy (MT) plus standard of care is superior to medical management alone in stroke patients with acute large vessel occlusion (LVO) (1). Up to 30% of patients with LVO may present with only minor clinical symptoms on admission (2). Evidence-based recommendations regarding the optimal management are lacking, as those patients were excluded from prospective trials or their number was markedly underrepresented (1, 3). Even if MT was shown to be effective irrespective of symptom severity on admission (1), observational studies still suggest a high risk of unfavorable outcome in those patients, mainly due to decompensating collaterals associated with secondary neurologic deterioration (4–6). Here, the occlusion site may be of importance (7). Nevertheless, only limited evidence from retrospective data exists regarding the efficacy of MT (8, 9), also in comparison to initial medical management with rescue intervention in case of secondary deterioration (10–12). However, mild stroke symptoms may not justify immediate MT in view of the procedure's invasiveness and potential serious adverse effects (13). Thus, patient selection may play a crucial role to identify patients with minor deficits and LVO who may benefit from an interventional treatment. In this retrospective study, we investigated characteristics, prognostic factors, and management of patients with LVO and minor clinical deficit on admission also in comparison to patients with moderate to severe deficits.

## Methods

### Patient Selection

This study was approved by our institutional review board (University of Erlangen-Nuremberg Re.-No. 377_17 Bc). We retrospectively identified all patients who (1) underwent MT due to an LVO [internal carotid artery (without T), intracranial internal carotid artery-T, M1 and M2 segment of the middle cerebral artery, basilar artery and vertebral artery] or (2) showed minor clinical deficits [defined as National Institutes of Health Stroke Scale (NIHSS) ≤ 4 on admission] due to an LVO (as defined above) irrespective of any acute stroke treatment (no reperfusion therapy, thrombolysis, MT, or both) between January 2013 and December 2016 from our prospectively organized institutional database. Patients had been admitted either to our tertiary stroke center or to one of our collaborating primary hospitals and were then transferred for MT (drip and ship). Clinical characteristics on admission, comorbidities and preadmission status, treatment and medication, symptom-to-groin time, and clinical parameters during the in-hospital stay were recorded.

### Patient Treatment

A trained stroke physician performed standardized clinical examination on admission and obtained the NIHSS. Neuroimaging was performed using computed tomography (CT) including CT angiography and CT perfusion or magnetic resonance imaging (MRI) including diffusion-weighted imaging, fluid attenuation inversion recovery, susceptibility weighted imaging, MRI angiography, and MRI perfusion to rule out an intracerebral hemorrhage, ensure LVO, and assess Alberta Stroke Program Early CT Score (14). Patients initially admitted to a primary care hospital were referred to our tertiary stroke center after diagnosis of LVO. Intravenous thrombolysis with recombinant tissue-type plasminogen activator was performed in accordance with recommendations of international guidelines (15, 16) due to the treating physician. The decision on MT was based on the agreement between the treating neurologist and the neurointerventionalist considering both clinical and imaging criteria.

### Assessment of Outcome

Outcome variables were intrahospital mortality, modified Rankin Scale (mRS) on day 90 [ “favorable outcome” (mRS 0–2) and “poor outcome” (mRS 3–6)] and secondary deterioration. Trained and certified physicians conducted phone interviews with patients or their next of kin to obtain day 90 mRS. Secondary deterioration was defined as an acute NIHSS increase of 4 or more points during the in-hospital stay (6). In patients with minor deficits, we assessed factors associated with outcome and secondary deterioration. In the MT cohort, we assessed factors associated with outcome also in relation to stroke severity on admission. Furthermore, we retrospectively divided patients with NIHSS ≤ 4 on admission into two groups for analysis: immediate mechanical thrombectomy (IT) or initial medical management with rescue intervention in case of secondary deterioration (MM) and correlated outcome with NIHSS on admission. If the decision to perform a thrombectomy was made in the context of the initial diagnostic workup, we defined this intention as “IT.” If it was decided not to perform a thrombectomy after the initial diagnostic workup, but this decision was revised after secondary deterioration, we defined it as “MM.” Furthermore, procedure-related complications and the occurrence of secondary intracerebral hemorrhage according to the ECASS-2 definition (17) and dichotomized final infarct size (greater than and less than one-third of corresponding artery territory) were investigated.

### Statistics

IBM^®^ SPSS^®^ Statistics 21 software package (IBM Corp, Armonk, NY) was used. The significance level was set at α = 0.05. Statistical tests were 2-sided. We used the Kolmogorov–Smirnov test to determine the distribution of data. Data were presented as mean and standard deviation (SD), as median and interquartile range (IQR, Mann–Whitney *U* test), or number (*n*) and percentage (Pearson χ^2^ or Fisher exact test), as appropriate. We used univariate logistic regression to calculate mortality-related odds ratios. Clinically meaningful parameters with *p* ≤ 0.1 in univariate testing were included in a multivariable logistic regression model for prediction of favorable outcome and secondary deterioration using stepwise backward inclusion (likelihood ratio). We excluded patients with missing outcome data from outcome-related analyses. For patients treated with MT, we performed an additional sensitivity analysis of functional outcome including only patients with anterior circulation LVO and a baseline mRS 0–2 (defined as mRS during the week before admission). In the minor stroke group, we performed an additional sensitivity analysis of factors associated with secondary deterioration excluding patients with IT.

## Results

### Patients' Characteristics and Outcome

Two hundred twenty-three patients treated with MT and 88 patients with minor deficits due to LVO were included (characteristics shown in Table 1). Thirteen patients with minor deficits due to LVO were treated with MT and thus were included in both groups. Functional outcome on day 90 was available in 89% of patients.

**Table 1:** Baseline characteristics of included patients.

**Characteristics**	**Patients with MT (*n* = 223)**	**Patients with minor stroke and LVO (*n* = 88)**
Age (IQR) (years)	75 (60–80)	67 (58–77)
Sex (female) (%)	120 (54)	22 (33)
Baseline mRS (IQR)	0 (0–2)	0 (0–1)
Hypertension (%)	180 (81)	74 (84)
Diabetes (%)	56 (25)	11 (13)
Hypercholesterinemia (%)	84 (38)	59 (67)
Renal insufficiency (%)	26 (12)	12 (14)
Atrial fibrillation (%)	113 (51)	25 (28)
Antiplatelet use (%)	186 (83)	32 (36)
Anticoagulation (VKA and DOAC) (%)	37 (17)	10 (11)
NIHSS on admission (IQR)	17 (13–21)	2 (1–3)
NIHSS 24 h (IQR)	25 (7–38; *n* = 200)	2 (1–4)
Secondary deterioration (acute NIHSS increase > 4 during in-hospital stay) (%)	15 (7, before MT)	19 (22)
MT (%)	223 (100)	13 (15)
Symptom to groin time (h)	5.8 (SD 7.4)	5.3 (IQR 9.1)
CT-ASPECTS on admission (IQR)	8 (7–9)	10 (9–10)
Intravenous thrombolysis (%)	171 (77)	15 (17)
Thrombolysis in primary care center (%)	61 (27)	3 (3)
**Location of vessel occlusion**		
Left-sided occlusion (anterior circulation) (%)	92 (50)	21 (45)
Internal carotid artery (without T) (%)	21 (9)	13 (15)
Intracranial internal carotid artery-T (%)	43 (19)	1 (1)
Middle cerebral artery: M1 (%)	107 (48)	14 (16)
Middle cerebral artery: M2 (%)	13 (6)	19 (22)
Posterior cerebral artery (%)	0 (0)	5 (6)
Vertebral artery (%)	3 (1)	25 (28)
Basilar artery (%)	36 (16)	11 (13)
**Outcome parameters**		
Infarct size greater than one-third of corresponding artery territory (%)	57 (26)	8 (9)
Symptomatic ICH (%)	7 (3)	2 (2)
Asymptomatic ICH (%)	17 (8)	0 (0)
Intrahospital mortality (%)	46 (21)	6 (7)
mRS on day 90 (IQR)	4 (2–6; *n* = 192)	2 (1–3)
Favorable outcome (mRS 0–2) (%)	54 (28; *n* = 192)	60 (68; *n* = 87)

*Data are given as mean and SD, median and IQR, and n (%) as appropriate. Thirty-one patients did not have day 90 outcome data available. VKA, vitamin K antagonist; DOAC, direct oral anticoagulant; ASPECT, Alberta Stroke Program Early CT Score; ICH, intracerebral hemorrhage; baseline mRS, mRS during the week before admission; minor stroke, NIHSS on admission ≤ 4*.

### Patients Treated With MT (*n* = 223)

Location of vessel occlusion did not differ between patients with favorable and poor outcome (χ^2^ = 9.879, *p* = 0.063). Multivariate logistic regression revealed an association of favorable functional outcome on day 90 with NIHSS on admission [odds ratio (OR), 0.951; 95% confidence interval (CI), 0.908–0.995; *p* = 0.028], pre-stroke mRS (OR, 0.554; 95% CI, 0.382–0.804; *p* = 0.002), dichotomized infarct size (OR, 0.049; 95% CI, 0.010–0.236; *p* < 0.0001), and a diagnosis of hypertension (OR, 0.273; 95% CI, 0.099–0.753; *p* = 0.012). Intravenous thrombolysis showed a trend toward a favorable outcome (OR, 3.067; 95% CI, 0.816–11.531; *p* = 0.097).

### Patients With LVO and NIHSS ≤ 4 on Admission (*n* = 88)

Sixty patients (68%) with LVO and minor deficits on admission had a favorable day 90 outcome. Higher baseline mRS, higher NIHSS on admission, secondary deterioration, M1 occlusion, and larger final infarct size were associated with poor functional outcome (Table 2). Multivariate logistic regression revealed baseline mRS (OR, 0.343; 95% CI, 0.163–0.722), initial NIHSS (OR, 0.496; 95% CI, 0.291–0.846), and secondary deterioration (OR, 0.060; 95% CI, 0.013–0.280) as independent predictors of poor functional outcome in patients with minor stroke and LVO on admission. Secondary deterioration was associated with higher baseline mRS, higher NIHSS on admission, and M1 occlusion, whereas vertebral artery occlusion was associated with a lower risk of secondary deterioration. An internal carotid artery occlusion was not associated with a secondary deterioration. An intracranial internal carotid artery-T occlusion was also not associated with a secondary deterioration in our cohort (Table 3). However, there was only one patient with ICA-T occlusion in our cohort, who was treated with IT. In the multivariate logistic regression, only M1 occlusion remained as a risk factor for secondary deterioration (OR, 29.9; 95% CI, 6.5–137.9). Comorbidities were associated neither with outcome nor with secondary deterioration. All three patients without secondary deterioration and M1 occlusion showed a reperfusion in control angiography. Two of them received intravenous thrombolysis in a primary stroke center and improved during transfer to our tertiary stroke center. One patient was treated with MT (IT group, see below). The sensitivity analysis without patients treated with IT (*n* = 80) revealed similar results while there was a trend toward an association of BA occlusion with secondary deterioration [4 (27%) patients with BA occlusion showed secondary deterioration, 5 (8%) did not, *p* = 0.058; further characteristics not shown]. However, all patients with secondary deterioration and BA occlusion showed a poor posterior communicating artery collateral flow and a large clot extent (*n* = 4), whereas in comparison patients without secondary deterioration and BA occlusion mainly showed a short proximal occlusion with a strong posterior communicating artery collateral flow [*n* = 5, *p* = 0.008; two patients were treated with IT (see below)].

**Table 2:** Characteristics of patients with minor deficits (NIHSS ≤ 4 on admission) and LVO stratified for outcome (favorable = day 90 mRS 0–2; unfavorable = day 90 mRS 3–6).

**Characteristics of patients with minor stroke (NIHSS on admission ≤ 4) and LVO (*n* = 88)**	**Favorable outcome (day 90 mRS 0–2; *n* = 60)**	**Unfavorable outcome (day 90 mRS 3–6; *n* = 28)**	***p*-value**
Age (IQR)^+^ (years)	67 (56–85)	73 (59–78)	0.251
Sex (female) (%)^*^	20 (33)	9 (32)	0.999
Baseline mRS (IQR)^+^	0 (0)	1 (0–3)	<0.0001
NIHSS on admission (IQR)^+^	2 (0–3)	3 (2–4)	<0.0001
NIHSS 24 h (IQR) ^+^	1 (0–2)	7 (3–34)	<0.0001
Secondary deterioration (NIHSS increase > 4 during in-hospital stay; %)^*^	3 (5)	16 (57)	<0.0001
Time between admission and secondary deterioration (h) (IQR)^*^	4 (1.7)	5.3 (2.5–24.3)	0.507
MT (%)^*^	3 (5)	10 (36)	<0.0001
Intravenous thrombolysis (%)^*^	8 (13)	7 (25)	0.225
**Location of vessel occlusion**			
Left-sided occlusion (anterior circulation) (%)^*^	14 (45)	7 (44)	0.995
Internal carotid artery (without T, %)^*^	11 (18)	2 (7)	0.212
Intracranial internal carotid artery-T (%)^*^	0 (0)	1 (4)	0.318
Middle cerebral artery: M1 (%)^*^	4 (7)	10 (36)	0.001
Middle cerebral artery: M2 (%)^*^	16 (27)	3 (11)	0.104
Posterior cerebral artery (%)^*^	5 (8)	0 (0)	0.173
Vertebral artery (%)^*^	18 (30)	7 (25)	0.800
Basilar artery (%)^*^	5 (18)	6 (10)	0.491
Infarct size greater than one-third of corresponding artery territory (%)^*^	0 (0)	8 (29)	<0.0001
Symptomatic ICH (%)^*^	1 (2)	1 (4)	0.999
Asymptomatic ICH (%)^*^	0 (0)	0 (0)	0.999

*Data are given as median and IQR and n (%) as appropriate*.

* *χ^2^/Fisher exact*.

+ *Non-parametric test (Wilcoxon rank-sum test). mRS, mRS during the week before symptom onset*.

**Table 3:** Characteristics of patients with minor stroke (NIHSS on admission ≤ 4) due to LVO stratified for secondary deterioration (acute NIHSS increase > 4 points during in-hospital stay).

**Characteristics [patients with minor stroke (NIHSS on admission ≤ 4) due to LVO, *n* = 88]**	**Secondary deterioration (acute NIHSS increase > 4; *n* = 19)**	**No secondary deterioration (*n* = 69)**	***p*-value**
Age (IQR)^+^ (years)	72 (64–77)	67 (56–76)	0.199
Sex (female) (%)^*^	6 (32)	23 (33)	0.999
Baseline mRS (IQR)^+^	1 (0–3)	0 (0–0.5)	0.014
NIHSS on admission (IQR)^+^	3 (2–4)	1 (0–3)	0.001
NIHSS 24 h (IQR) ^+^	8 (5–20)	2 (0–3)	<0.0001
MT (%)^*^	9 (48)	4 (6)	<0.0001
Intravenous thrombolysis (%)^*^	6 (32)	9 (13	0.083
**Location of vessel occlusion**			
Left-sided occlusion (anterior circulation) (%)^*^	8 (57)	13 (39)	0.531
Internal carotid artery (%)^*^	1 (5)	12 (17)	0.283
Intracranial internal carotid artery-T (%)^*^	0 (0)	1 (1)	0.999
Middle cerebral artery: M1 (%)^*^	11 (58)	3 (4)	<0.0001
Middle cerebral artery: M2 (%)^*^	2 (11)	17 (25)	0.226
Posterior cerebral artery (%)^*^	0 (0)	5 (7)	0.352
Vertebral artery (%)^*^	1 (5)	24 (35)	0.019
Basilar artery (%)^*^	4 (21)	7 (10)	0.242
Infarct size greater than one-third of corresponding artery territory (%)^*^	7 (37)	1 (1)	<0.0001

*Data are given as median and IQR and n (%) as appropriate*.

* *χ^2^/Fisher exact*.

+ *Non-parametric test (Wilcoxon rank-sum test). baseline mRS, mRS during the week before symptom onset*.

### Patients With LVO Treated With MT: NIHSS ≤ 4 on Admission (*n* = 13) vs. NIHSS > 4 (*n* = 210)

**Figure 1:**
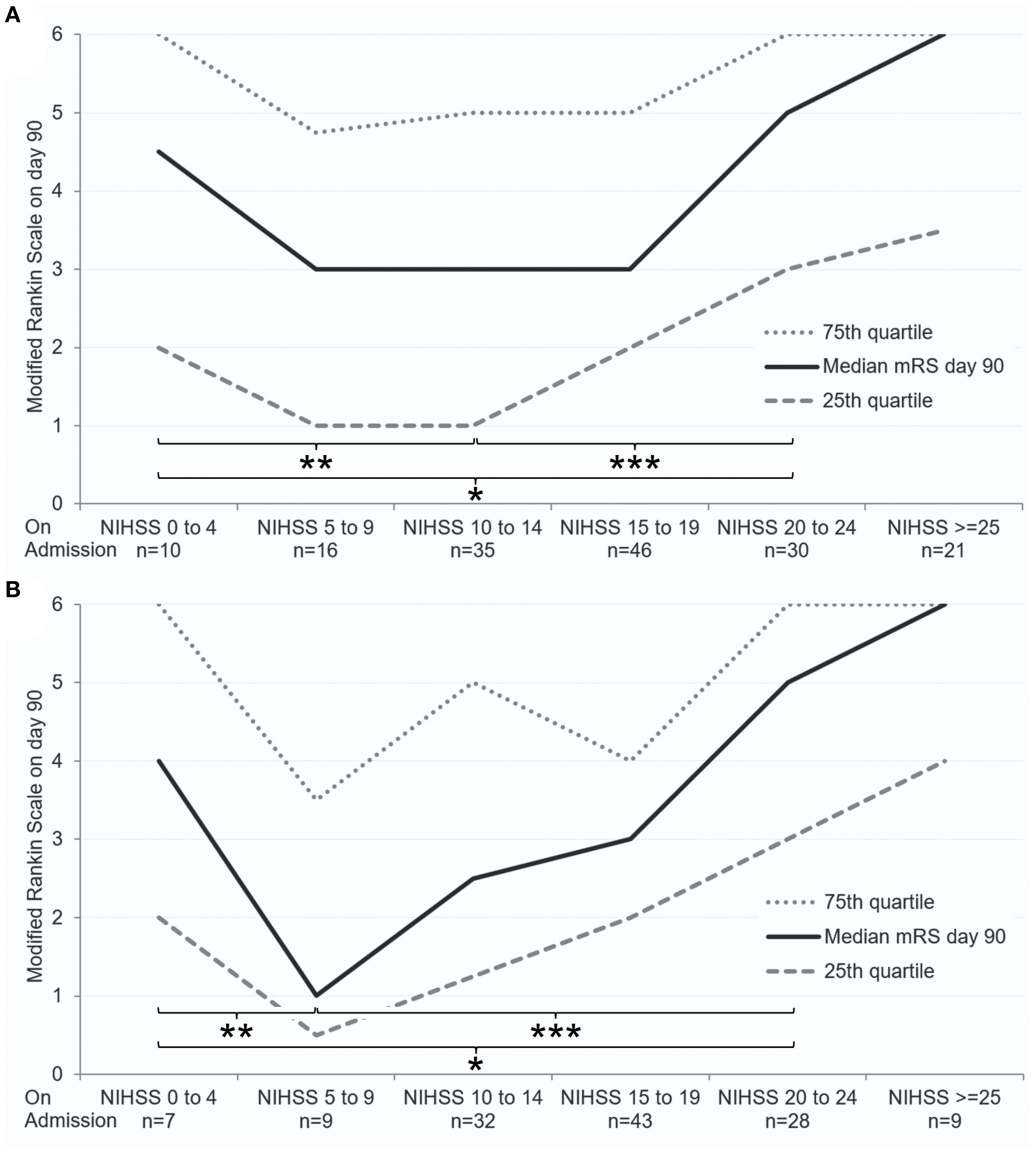
Association of NIHSS on admission with functional outcome on day 90 in patients treated with MT. Only patients with baseline mRS 0–2 (during the week before admission) were included in this figure. **(A)** Including anterior and posterior circulation strokes (*n* = 158). **(B)** Including only anterior circulation strokes (cohort of sensitivity analysis, *n* = 154; 26 patients did not have day 90 outcome data). Median mRS (black line) and 25th and 75th quartiles (dotted gray lines) are displayed. ***p < 0.05, **p < 0.1, *p > 0.1 (Wilcoxon rank-sum test between indicated patient groups).

Thirteen patients with NIHSS ≤ 4 on admission and LVO received MT (Table 4). Compared to patients with more severe deficits (NIHSS > 4), patients with low NIHSS scores on admission were more likely to be female. They also showed a trend toward a lower age and lower thrombolysis rates. There were no differences between both groups regarding the site of vessel occlusion (χ^2^ = 2.668, *p* = 0.705), comorbidities, medication, and secondary intracerebral hemorrhage rate (Table 4). The established association of symptom severity on admission (as quantified using the NIHSS score) with functional day 90 outcome (1) seems to inverse in treated patients with mild symptoms (NIHSS ≤ 4, Figure 1A) compared to moderately and severely affected stroke patients. Our sensitivity analysis showed consistent results (*n* = 154, characteristics not shown, Figure 1B): Patients with only minor neurologic deficits on admission despite an LVO treated with MT seem to have a worse functional day 90 outcome than patients with moderate stroke severity at baseline.

**Table 4:** Characteristics of patients treated with MT with NIHSS ≤ 4 and >4 on admission.

**Characteristics (patients treated with MT; *n* = 223)**	**NIHSS on admission ≤ 4 (*n* = 13)**	**NIHSS on admission > 4 (*n* = 210)**	***p*-value**
Age (IQR)^+^ (years)	65 (58–75)	75 (62–81)	0.056
Sex (female) (%)^*^	2 (16)	118 (56)	0.004
Baseline mRS (IQR)^+^	1 (0–2.5)	0 (0–2)	0.549
NIHSS on admission (IQR)^+^	4 (2.5–4)	17 (13.8–22)	<0.0001
Symptom to groin time (h) (IQR)^+^	5.3 (3.6–12.6)	3.9 (2.4–6.4)	0.169
CT-ASPECTS on admission (IQR)^+^	9 (7.5–10)	8 (7–9)	0.410
Left-sided occlusion (anterior circulation) (%)^*^	5 (50)	87 (50)	0.999
Intravenous thrombolysis (%)^*^	7 (54)	164 (78)	0.082
Intrahospital mortality (%)^*^	5 (39)	41 (20)	0.149
mRS on day 90 (IQR; *n* = 191)^+^	4.5 (2.5–6)	4 (2–6)	0.326
Favorable outcome (mRS 0–2) (%; *n* = 191)^*^	3 (25)	51 (29)	0.999
Symptomatic ICH (%)^*^	1 (8)	6 (3)	0.347
Asymptomatic ICH (%)^*^	0 (0)	17 (8)	0.606
Procedure-related complications (%)^*^	0 (0)	21 (10)	0.377
Infarct size greater than one-third of corresponding artery territory (%)^*^	4 (31)	53 (25)	0.744

*Data are given as median and IQR and n (%) as appropriate. Thirty-two patients did not have day 90 outcome data available*.

* *χ^2^/Fisher exact*.

+ *Non-parametric test (Wilcoxon rank-sum test). baseline mRS, mRS during the week before symptom onset; ASPECTS, Alberta Stroke Program Early CT Score*.

### Immediate vs. Rescue Thrombectomy in Patients With LVO and NIHSS ≤ 4 on Admission (*n* = 13)

Thirteen patients with minor deficits and available outcome data were dichotomized to either IT (eight patients, IT) or initial medical management with rescue intervention after secondary clinical deterioration (five patients, MM). Relevant clinical and demographic characteristics (Table 5) and proportion of posterior circulation stroke did not differ between the IT and MM groups [2 (25%) and 1 (20%), *p* = 0.999]. Four patients in the IT group showed a secondary deterioration after decision to perform IT was made. Median time between admission and secondary deterioration was 6.8 h (IQR, 3.3–21.2) in the MM group, and median time between secondary deterioration and groin was 1.1 h (IQR, 0.8–1.7). IT was associated with a lower intrahospital mortality (OR, 0.036; 95% CI, 0.002–0.741) compared to MM (13 vs. 80%, *p* = 0.032). Twenty-nine percent of patients with available outcome data treated with IT had a favorable outcome on day 90.

**Table 5:** Characteristics of patients with NIHSS on admission ≤ 4 (minor stroke) who were treated with MT dichotomized according to IT and MM.

**Characteristics [patients with minor stroke (NIHSS on admission ≤ 4) who were treated with MT, *n* = 13]**	**IT group, *n* = 8**	**MM group, *n* = 5**	***p*-value**
Age (IQR)^+^ (years)	60 (56–72)	70 (66–76)	0.106
Sex (female) (%)^*^	0 (0)	2 (40)	0.128
Baseline mRS (IQR)^+^	0 (0–2.75)	1 (0.5–2.5)	0.438
NIHSS on admission (IQR)^+^	4 (3–4)	3 (1–4)	0.135
Secondary deterioration (NIHSS increase > 4 before thrombectomy; %)^*^	4 (50)	5 (100)	0.105
NIHSS before thrombectomy (IQR)^+^	6 (4–11)	15 (10–18)	0.019
Symptom to groin time (h) (IQR)^+^	3.9 (2–5)	13.9 (9–24.5)	0.030
CT-ASPECTS on admission (IQR)^+^	8.5 (7–10)	9 (7–10)	0.943
Intravenous thrombolysis (%)^*^	5 (62)	2 (40)	0.592
Intrahospital mortality (%)^*^	1 (13)	4 (80)	0.032
mRS on day 90 (IQR; *n* = 12)^+^	4 (2–5)	6 (4–6)	0.148
Favorable outcome (mRS 0–2) (%; *n* = 12)^*^	2 (29, n = 7)	1 (20)	0.999
Symptomatic ICH (%)^*^	1 (13)	0 (0)	0.999
Asymptomatic ICH (%)^*^	0 (0)	0 (0)	0.999
Procedure-related complications (%)^*^	0 (0)	0 (0)	0.999
Infarct size greater than one-third of corresponding artery territory (%)^*^	2 (25)	2 (40)	0.999

*Data are given as median and IQR and n (%) as appropriate. One patient did not have day 90 outcome data available*.

* *χ^2^/Fisher exact*.

+ *Non-parametric test (Wilcoxon rank-sum test). baseline mRS, mRS during the week before symptom onset; ASPECTS, Alberta Stroke Program Early CT Score*.

## Discussion

In this observational study, patients with minor neurological deficits on admission despite LVO had a high risk of poor outcome, if secondary deterioration occurred. In those patients, rescue endovascular thrombectomy was associated with a poor outcome in our cohort. Secondary deterioration seems to be associated with occlusion site and associated factors, which may guide selection criteria for future RCTs to identify patients who may benefit from revascularization.

Until present, it remains unclear whether patients with LVO and minor clinical deficits on admission would benefit from IT compared to primarily medical management. The scenario of active collaterals that initially attenuate the hemodynamic effect of LVO applies for up to 30% of acute stroke patients with LVO and is associated with a high risk of poor outcome, mainly due to secondary deterioration (2, 5, 18). The major thrombectomy trials did not focus on those patients sufficiently (1, 3), and retrospective data revealed controversial results (9, 10, 12).

In our study, the group of patients with NIHSS ≤ 4 on admission and LVO treated with MT seems to show a disproportionately poor outcome compared to patients with moderate or severe deficits, especially in cases with initial medical management and secondary deterioration. Even if these findings support existing data regarding the high risk of unfavorable outcome in those patients, they also contradict the established efficacy of MT irrespective of the clinical status on admission (1, 3). As we found low rates of symptomatic secondary hemorrhage in those patients (13), we consider the fatal clinical course of patients with initial medical management, which was associated with a large final infarct size, as possible explanation. Accordingly, the proportion of patients with large final cerebral infarction did not differ between patients with minor and moderate–severe stroke severity on admission. Especially, patients suffering secondary deterioration showed a higher rate of a large final cerebral infarction size. Therefore, the severity of the condition should not be underestimated on admission. Even patients with minor deficits on admission may need an appropriate diagnostic in the acute phase including vessel imaging to rule out an LVO. Thus, our results underline the well-established concept of early reperfusion therapy in stroke in general and the detrimental consequences of a secondary clinical worsening (1, 5). A limitation of this interpretation may be seen in certain imbalances between patients with minor stroke and patients with moderate to severe deficits in our cohort: There was a lower proportion of females in the minor stroke group, which may not explain the results, but should be kept in mind when interpreting our results. The trend toward a lower thrombolysis rate in our cohort treated with MT may also be associated with a poor outcome, whereas on the other hand, the trend toward a younger age in those patients may be associated with a favorable outcome (1). Because of the small sample size, we could not adjust for those variables in our analysis.

Furthermore, symptom onset to groin time was longer in patients treated with rescue MT. In the DAWN trial as well as in the DEFUSE-3 trial, a benefit of thrombectomy was found in patients up to 24 and 16 h after symptom onset, respectively (19, 20). However, both trials used strict selection criteria including a prominent clinical-core mismatch with an NIHSS of more than 10 prior to inclusion and perfusion imaging, respectively. Furthermore, patients with minor stroke were neither included in DAWN nor in DEFUSE-3 irrespective of secondary deterioration.

However, strict selection criteria may also play a crucial role to identify patients with minor deficits due to LVO who might benefit from thrombectomy or thrombolysis. In our minor stroke cohort, intravenous thrombolysis was not associated with outcome. Although we did not assess this association further, existing results have shown that thrombolysis may be associated with potential harm in patients presenting with an NIHSS score of 0–1 compared to patients with a score of 2–5 (21). Other results suggested that intravenous thrombolysis might also be associated with deterioration due to thrombus fragmentation in some patients with ICA occlusion (22). The efficacy of bridging thrombolysis in those patients also remains to be elucidated (23). Those results underline the necessity to select the right patient for the right treatment. Secondary deterioration seems to be strongly associated with outcome (6), which we could also show in our cohort. In turn, secondary deterioration seems to be associated with the occlusion site (6). Here, our results suggest an M1 occlusion as a strong predictor, whereas a vertrebral artery occlusion does not seem to be associated with secondary deterioration. Seners et al. found that in anterior circulation stroke patients with M2 occlusion had a lower risk of secondary deterioration than patients with M1 occlusion or carotid artery-T occlusion. It seems that the more distal the occlusion site could be found in patients with minor stroke, the lower was the associated risk of secondary deterioration (6). As we could include only one patient with carotid artery-T occlusion in our minor stroke cohort and did not differentiate distal from proximal M1 occlusions, we could not assess dedicated ORs for carotid artery-T occlusions and different M1 occlusion sites. Furthermore, the patient with carotid artery-T occlusion was treated with IT; thus, no conclusions regarding the prognostic value of carotid-t occlusions to predict secondary deterioration can be drawn from our data. However, in general, our data also support these findings that proximal middle cerebral artery occlusions are associated with higher risks of secondary deterioration than distal ones. On the other side, we found no association of internal carotid artery occlusions with secondary deterioration in patients with minor deficits, with only one patient (5%) showing a secondary deterioration. None of the patients with internal carotid artery occlusion and minor deficits on admission received MT; only one patient received thrombolysis (data not shown). Still 11 patients showed a favorable outcome. This raises the question whether patients with a mere internal carotid artery occlusion and minor deficits on admission should be treated with MT. As we could not assess collateral status and could include only a small number of those patients, our data cannot sufficiently answer this question, which should be addressed in future research. For patients with basilar artery occlusion, clot extent and collaterals seem to play a special role. However, because of small cohort size and a rather qualitative assessment, those results should be interpreted with care. Furthermore, our results suggest an association of the severity of clinical symptoms with deterioration even within the group of patients with minor deficits as defined, although we could not show this association in our multivariable logistic regression model any more. Again, because of the small cohort size, those results also have to be interpreted with care. Those factors may play a role when defining inclusion criteria for future RCTs to assess the efficacy of a revascularization treatment in patients with minor deficits due to LVO.

There are several limitations to our study: The retrospective single-center design and the limited number of patients with mild clinical symptoms on admission treated with MT might compromise the generalizability of our results. Furthermore, comparative statistics should be interpreted with caution. Our cohort consisted of patients with LVO in both the anterior and posterior cerebral circulation. However, our sensitivity analysis showed consistent results. In the IT group, some patients showed neurologic deterioration before thrombectomy. However, treatment was not delayed, and the decision to perform MT was made before deterioration. During the study period, several influential trials on MT (1) have been published, which may have altered clinical practice. However, patients with minor deficits have not sufficiently been addressed in those trials. In accordance with others (24, 25), we defined minor stroke using an NIHSS score < 5. Other NIHSS cutoff values have been described to define a minor stroke, which may have altered our results. We did not perform a quantitative assessment of clot extent/thrombus length and collateral flow in patients with BA occlusion, which may limit our results. Also, no assessment of collateral flow could be performed in anterior circulation LVOs. This assessment was performed only comparatively. However, the small number of patients included for this assessment did not justify an elaborate quantitative assessment.

## Conclusion

Patients with mild neurologic deficits on admission despite a large vessel occlusion might have a high risk of unfavorable outcome and a large final infarct size especially in cases of secondary deterioration. Initial medical management with rescue intervention after clinical deterioration was associated with a fatal clinical course in our cohort. Occlusion site and associated factors showed an association with secondary deterioration, which may guide selection of patients for clinical trials. Quick diagnosis is essential, and immediate recanalization of LVO may be addressed in future RCTs even in patients with minor stroke, if adequate inclusion criteria are applied.

## Data Availability Statement

The raw data supporting the conclusions of this article will be made available by the authors, without undue reservation.

## Ethics Statement

The studies involving human participants were reviewed and approved by Ethikkommission der Friedrich-Alexander-Universität Erlangen-Nürnberg Krankenhausstraße 12 91054 Erlangen. Written informed consent for participation was not required for this study in accordance with the national legislation and the institutional requirements.

## Author Contributions

BV, RG, TE, AM, KM, SS, AD, SL, and BK: acquisition, analysis and interpretation of data for the work, revising the manuscript, and final approval of the version to be published. BV, SL, and BK: conception and design of the work and drafting the work. All authors contributed to the article and approved the submitted version.

## Conflict of Interest

The authors declare that the research was conducted in the absence of any commercial or financial relationships that could be construed as a potential conflict of interest.

## Publisher's Note

All claims expressed in this article are solely those of the authors and do not necessarily represent those of their affiliated organizations, or those of the publisher, the editors and the reviewers. Any product that may be evaluated in this article, or claim that may be made by its manufacturer, is not guaranteed or endorsed by the publisher.
